# Segmental Electroacupuncture for Uterine Fibroids: A Case Report

**DOI:** 10.1002/ccr3.72011

**Published:** 2026-02-16

**Authors:** Elham Hooshyarazar, Hoda Azizi, Roghayeh Yahyazadeh

**Affiliations:** ^1^ Department of Acupuncture, School of Persian and Complementary Medicine Mashhad University of Medical Sciences Mashhad Iran; ^2^ Student Research Committee, School of Persian and Complementary Medicine Mashhad University of Medical Sciences Mashhad Iran; ^3^ Smart University of Medical Sciences Tehran Iran; ^4^ Immunology Research Center Ardabil University of Medical Sciences Ardabil Iran

**Keywords:** acupuncture, complementary therapies, electroacupuncture, integrative medicine, leiomyoma, uterine fibroids

## Abstract

Uterine myomas, benign genital tract tumors, commonly lead to troublesome symptoms in females of reproductive age. Despite various surgical and hormonal treatments available for myomas, their possible side effects warrant the exploration of complementary medicine for myoma management. Segmental electroacupuncture on acupoints located on myotomes with the same innervation as the uterus was performed for a patient diagnosed with myoma and menorrhagia, twice a week for 2 months in the acupuncture clinic of Imam Reza Hospital in Mashhad, Iran. Transvaginal ultrasound revealed a reduction in the size of the myoma and ovarian cyst. Patient's heavy menstrual bleeding, clot passing and fatigue associated with the myoma decreased after 2 months of treatment and her quality of life adversely affected by myoma‐related symptoms, improved. Electroacupuncture applied to acupoints that have the same innervation as the uterus has been shown to alleviate menorrhagia and dysmenorrhea associated with myomas, and it may serve as a safe substitute for conventional therapies for symptomatic myomas with low invasiveness and no adverse effects while preserving female fertility.

## Key Clinical Message

- Segmental electroacupuncture can alleviate myoma‐related symptoms with low invasiveness, no adverse effects, and the advantage of preserving female fertility.
- Limited number of needles utilized in this technique makes it an ideal option for individuals with needle phobia or those whose body areas are not entirely accessible to perform traditional acupuncture.

## Graphical abstract

Case report of a uterine myoma treated by segmental electroacupuncture.

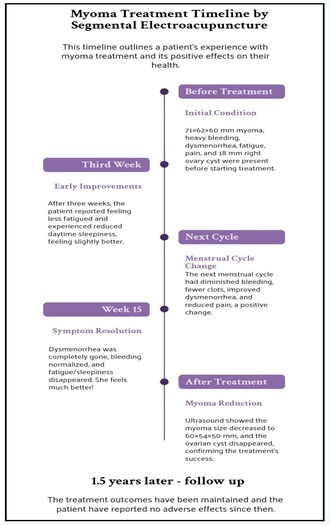

**Abbreviations**
CT scan: computed tomography scan
EA: electroacupuncture
HPO axis: Hypothalamic–Pituitary–Ovarian axis
Hz: hertz
mm: millimeter

## 1 Introduction

Uterine myomas, prevalent benign tumors within the realm of gynecological disorders, predominantly originate from the proliferation of smooth muscle cells in the uterine tissue [1]. These tumors, which mainly manifest themselves by abnormal uterine bleeding, pelvic pressure and related symptoms, and fertility dysfunctions, affect approximately 20%–40% of females within the reproductive age group [2]. They serve as the primary reason for performing hysterectomies across the globe and are correlated with a considerable psychosocial and financial impact on both the patient and health care systems [3].

The formation of myomas primarily relies on elevated levels of estrogen and progesterone. Consequently, their incidence is rare before menarche and after menopause due to lower estrogen levels in these age groups [4]. Several other elements are reported to be implicated in the formation of fibroids, including age, family history, race and ethnicity, parity, prolonged hormonal exposure, obesity, genetic changes, growth factors, neuropeptides, and dietary habits [5].

Numerous imaging techniques exist for the examination of myomas: ranging from hysterosalpingography (with very low sensitivity and specificity) to transabdominal ultrasound, sonohysterography, transvaginal ultrasound (known for higher sensitivity and specificity), MRI (the most accurate imaging modality) and CT scan [6].

Effective treatments of myomas rely on the size, location, and growth pattern of the tumor, as well as associated symptoms [7]. Currently, a variety of therapeutic approaches are employed for the management of myomas, which fall into two main categories: surgical interventions and non‐surgical methods, classified into two significant types: hormonal and non‐hormonal drugs. While all these approaches are recognized for their beneficial and significant impact on the treatment of myomas, various adverse effects have been documented in studies, including liver damage, androgenic effects, hot flashes, and decreased bone mineral density, along with risks of recurrence, uterine rupture, and postoperative adhesions in surgical interventions [8, 9].

Acupuncture, used in the management of numerous disorders worldwide, can exert a regulative effect on the endocrine and the central nervous systems; therefore, it could be proposed as a promising treatment option for myomas [10].

Electroacupuncture (EA) integrates acupuncture with electrical stimulation applied to acupoints, offering the advantage of amplifying acupuncture's effects and is extensively utilized in the management of various diseases. Segmental acupuncture relies on the concept of segmental anatomy, which originates from a spinal nerve and extends to the skin, providing branches to muscles, bones, and viscera. The selection of acupoints is determined based on the relevant segments associated with the affected organs, which are supplied by a single spinal nerve.

This article documents a case of a 46‐year‐old woman who presented with uterine myoma in December 2023, which caused her monthly debilitating menorrhagia with large clots and dysmenorrhea.

## 2 Clinical Report

This is a case report of a 46‐year‐old woman with a history of one pregnancy, diagnosed with uterine myoma. The myoma caused her monthly debilitating and heavy menstrual bleeding with large clots and dysmenorrhea just before the beginning of her menstrual cycle and during the first 3 days of her menses, leading to her absence from work for 1 to 2 days. The pain was in her lower back and lower abdomen, radiating down to the medial parts of her legs. She also felt fatigued and less productive, and the symptoms interfered with her physical activities, causing her to feel drowsy during the day. Due to the patient's proficiency in English, she was asked to fill out the English version of the UFS‐QOL (Uterine Fibroid Symptom and health‐related Quality of Life) questionnaire, which is a validated and standardized patient‐reported outcome measure designed to evaluate fibroid‐related symptoms. This questionnaire consists of two main sections: (1) Symptom Severity section, where higher score values are indicative of greater symptom severity; and (2) Health‐Related Quality of Life (HRQL) section, where higher scores will be indicative of better quality of life and lesser impact of uterine fibroids on the patient's quality of life [11]. Our patient's symptom severity transformed score appeared to be 62.5, and her HRQL transformed score was 45.68. The patient reported no prior history of abortions, did not smoke cigarettes, and avoided alcohol consumption or taking oral contraceptives. Moreover, there was a positive family history of myomas in her paternal relatives.

Transvaginal ultrasound evaluation on the seventh day of the patient's menstrual cycle revealed a sub‐serosal pedunculated myoma located on the posterior‐medial wall inclined to the right. The dimensions of the myoma were 71 × 62 × 60 mm, and the uterine body measured 81 × 36 mm. There was an 18 mm simple cyst in the right ovary. She expressed a strong aversion to receiving surgical or hormonal treatments for uterine myoma, driven by her fear of surgical operations and apprehension regarding the possible adverse effects associated with hormonal medications. As a result, she decided to seek acupuncture for relief from the symptoms associated with the myoma.

## 3 Treatment Course

**FIGURE 1:**
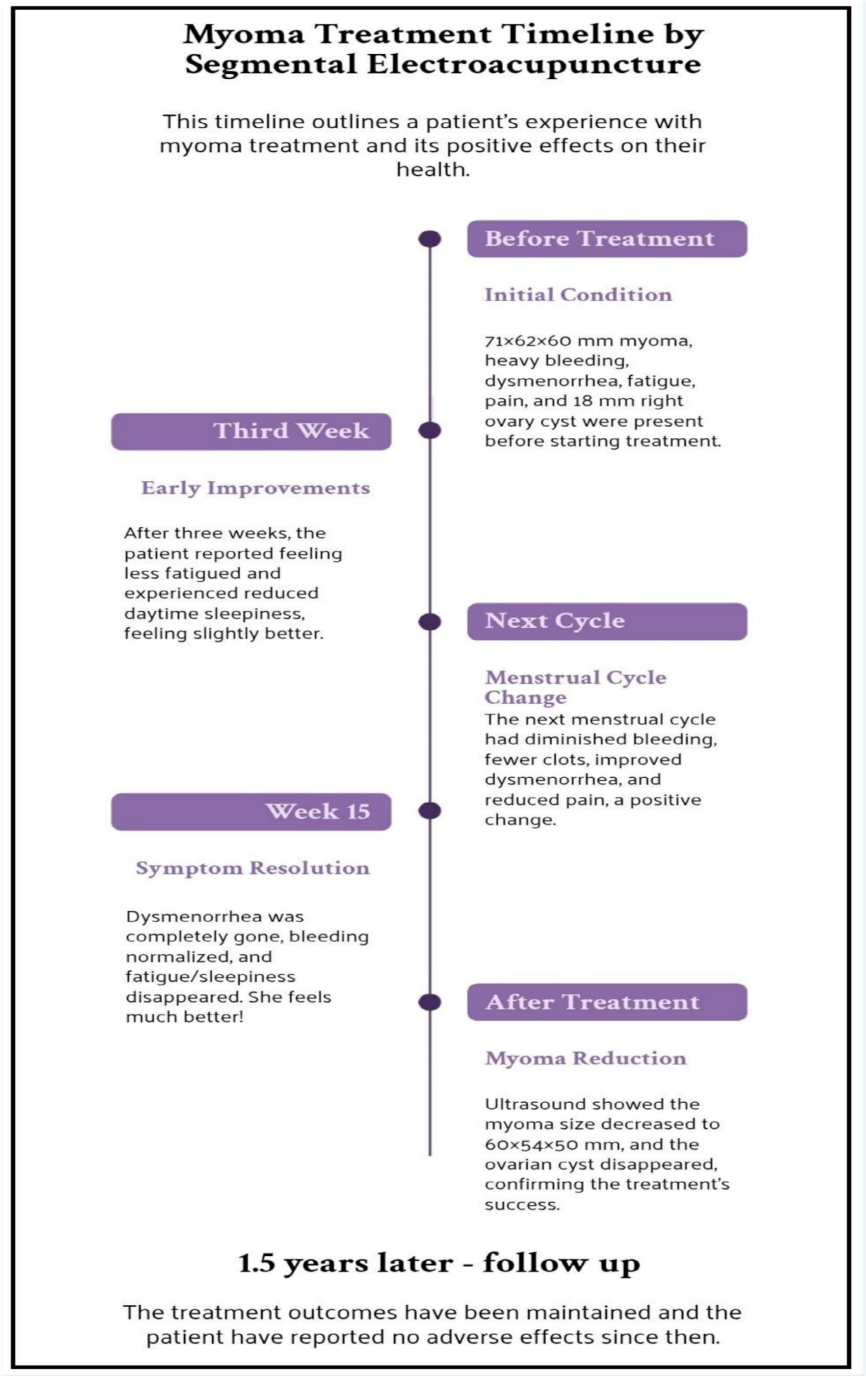
Patient treatment timeline.

The treatment course took place in the acupuncture clinic of Imam Reza Hospital in Mashhad, Iran. Figure 1 depicts a brief outline of the treatment course. We informed the patient about some possible adverse effects associated with acupuncture, such as insertion site bruising. Following the assurance that gynecological treatment would be provided immediately and free of charge in case of symptom exacerbation, the patient provided written informed consent for participation in this intervention and publication of associated clinical data. We initiated the EA treatment with the aim of reducing uterus and myoma size along with menorrhagia and dysmenorrhea stemming from myoma. Single‐use sterile stainless‐steel acupuncture needles (0.25 mm × 40 mm, Huan‐Qiu, Suzhou Huanqiu Acupuncture Medical Appliance Co. Ltd., China) were used for the procedure. After disinfecting the needle insertion area with 70% ethanol alcohol pads, the needles were inserted at specific acupoints on the abdomen (ST28 Shuidao and ST29 Guilai) and lower limbs (SP6 Sanyinjiao and LR3 Taichong), which are located within myotomes with the same innervation as the uterus, and de qi sensation was provoked. The handles of the needles were connected to the EA device (KWD‐8081, Chengzhou Yingdi Electronic Medical Device Co. Ltd), pairing the needles on the abdomen together and the needles on the lower limbs together unilaterally. The total time of EA intervention was 30 min for each session. The current intensity started from 2 mA and increased to the level that the patient didn't report pain. Treatment was conducted twice a week for 15 sessions over 9 weeks, from January 9, 2024 to March 5, 2024. The patient's symptoms were checked and evaluated before each treatment session. At the third week of treatment, the patient reported experiencing less fatigue and reduced daytime sleepiness. The onset of her next menstrual period occurred at the expected time without any discomfort, and her heavy menstrual bleeding, along with the amount and size of the blood clots, diminished, which subsequently lowered the number of pads she needed to change. Dysmenorrhea and abdominal cramps showed improvement. However, she still felt mild pelvic and back pain, prompting us to continue the treatment plan. By the 15th session of EA, not only had her dysmenorrhea completely gone, but her clot passing and heavy bleeding had also reduced significantly, resulting in a return to normal menstruation. Meanwhile, her fatigue and sleepiness during the day were no longer present, which empowered her to undertake social and physical activities. Based on the patient's responses obtained from the UFS‐QOL questionnaire she filled out immediately after the 15th session, her symptom severity transformed score dropped to 15.62, and her HRQL transformed score rose to 73.27. Transvaginal ultrasound measurements conducted following the last treatment session indicated that the myoma size had diminished to 60 × 54 × 50 mm and the ovarian cyst had disappeared. To maintain the accuracy of objective outcome measurements, the ultrasound measurements were done by a single radiologist using the same equipment in the same clinic in both pre‐ and post‐treatment scans.

At the time of writing, the outcomes of this intervention had been maintained for a year and a half, and our patient had reported no adverse effects since then. We asked her to fill out the UFS‐QOL questionnaire at that time point as a follow‐up evaluation, and the results turned out to be completely satisfying. Her symptom severity transformed score declined to 6.25, and the HRQL transformed score increased to 81.89, which demonstrated the sustainability of intervention effects in the long run.

## 4 Discussion

In this case, we provide evidence that segmental EA can alleviate troublesome symptoms related to myoma, such as heavy menstrual bleeding, passing clots, fatigue, and dysmenorrhea. Furthermore, transvaginal ultrasound findings indicated a reduction in myoma size and the disappearance of the ovarian cyst. This was the first case in the literature to perform segmental acupuncture on myotomes sharing the same innervation as the uterus. The segmental approach is used if direct needling of the target tissue is impractical. This is most applicable in the treatment of visceral conditions.

As afferent pathways from the viscera and from somatic structures (skin and muscles) merge into a unified pathway at the dorsal horn, transmitting one type of signal to the brain, irrespective of its somatic or visceral source, acupuncturists can take advantage of this convergence to influence the organ by needling the muscle that shares the same segmental innervation. The input that each segment receives, from either the body organs or the brain, causes a modification in its activity. Acupuncture stands as one input that can influence the spinal cord's management of the autonomic reflex. Furthermore, afferent information can impact the higher supraspinal centers, which then affects the segment‐specific autonomic reflex (descending autonomic control). This can effectively restore normal function to organs receiving nerve supply from that segment through the autonomic nervous system. Intramuscular needling is recognized as the standard method for evoking autonomic responses. Acupuncture generally operates through the nerves within muscles; thus, myotomes are considerably important when employing segmental acupuncture [12].

In past years, the effectiveness and safety of acupuncture for the management of myomas were reported as being uncertain [10, 13]; however, most recent systematic reviews in the field demonstrated that the feasibility, efficacy, and safety of various methods of acupuncture (traditional acupuncture, electroacupuncture, warm acupuncture, moxibustion, and acupoint catgut embedding) were superior to the control groups (Chinese herbal medicine, mifepristone), especially in myoma size reduction, with no serious adverse events [14]. Moreover, it has been shown that acupuncture combined with Chinese herbal medicine is more effective than Chinese herbal medicine alone in reducing myoma size and hormone levels, along with improving myoma‐related symptoms and quality of life [15].

Studies prove that myoma development greatly depends on a feedback loop between female sex hormones and growth factors. Regarding the fact that acupuncture can regulate the endocrine system and the neuroendocrine system, such as the hypothalamic–pituitary–ovarian (HPO) axis, which is linked to myoma growth, acupuncture would be an appropriate holistic therapy for myomas, with minimal invasion and fewer side effects if administered by qualified experts in the field [10, 14, 16, 17]. Previous studies have supported the effectiveness of traditional acupuncture treatment for myomas [18, 19, 20, 21, 22, 23]. Evidence reveals that acupuncture can modulate various factors and theoretical perspectives linked to the development and clinical symptoms of uterine fibroids, such as neuroendocrine and hormonal pathways, growth factors, oxidative stress, inflammation, and uterine blood flow [24].

Clinical experience demonstrates that initiation of restricted blood flow in the uterine arteries, whether performed with a clamp or via uterine artery embolization, effectively leads to a decrease in the size of fibroids and alleviates menorrhagia [25, 26, 27]. Research has shown that EA exhibits a remarkably selective effect on increasing or decreasing blood flow to a target organ, depending on the appropriate combinations of nerve stimulation and frequency. Moreover, only high‐frequency EA has been proven to reduce ovarian blood flow in rats; and in addition to segmental innervation, central effects are also believed to be involved in this process, because even after the ovarian sympathetic nerves were cut, the ovarian blood flow responses to high‐frequency EA were still observed [28, 29]. According to a case report carried out in Turkey, EA with 80 Hz frequency and 180 microseconds wavelength, by making a specific combination of stimulation frequency and a dermatome—which has been proven to decrease ovarian blood flow in rats—showed a decrease in blood flow of the uterine arteries and improved the amount of menorrhagia due to a submucous myoma [29]. Acupuncture can be introduced as a beneficial therapeutic option, as revealed by this and other studies, for patients who prefer nonsurgical or non‐pharmacological treatments for myomas.

Our case study has some limitations. Initially, hemoglobin levels, female sex hormone profiles, and growth factors that significantly influence myoma growth, as well as the blood flow parameters of the uterine arteries responsible for supplying blood to the myoma, were not assessed in response to the different frequencies of the EA applied to specific body regions that share the same innervation as the uterus. Further investigations are essential to assess the effects of EA on hemoglobin, hormone and growth factor levels, and myoma blood supply indicators in a more detailed method to clarify the mechanism of action of this novel acupuncture protocol. Secondly, utilizing a minimum number of needles in this method makes it a cost‐effective, affordable protocol favorable for patients with a fear of needles; however, this study, as a single‐case report, lacks a comparative intervention or a control group. Thus, the improvements that were observed cannot be definitively attributed to the acupuncture treatment alone and may be affected by factors such as placebo effects. On the other hand, as our findings are based on the clinical progression of a single patient, they lack the generalizability of larger cohort or controlled investigations. The observations presented here, while offering valuable clinical insights and highlighting a novel treatment approach, should be regarded as hypothesis‐generating rather than definitive. Therefore, the effectiveness of this method should be verified in additional well‐designed comparative studies, such as randomized controlled trials with sham acupuncture, in the future on a larger number of patients to establish its broader applicability. A randomized controlled trial incorporating the aforementioned characteristics is currently in progress [30].

## 5 Conclusion

This case indicates that segmental EA may be a safe and effective option for managing symptoms related to myomas, with the advantage of preserving women's fertility. The limited number of needles utilized in the segmental electroacupuncture method makes it an ideal acupuncture option for individuals with needle phobia or for those whose body areas are not entirely accessible for performing traditional acupuncture due to surgical scars or dermatological diseases. However, larger and more rigorous clinical trials with control groups are necessary to confirm these results and determine the exact role of segmental EA in myoma management.

## Author Contributions

**Elham Hooshyarazar:** conceptualization, data curation, formal analysis, investigation, methodology, project administration, resources, software, validation, visualization, writing – original draft, writing – review and editing. **Hoda Azizi:** conceptualization, funding acquisition, investigation, methodology, project administration, resources, supervision, validation, visualization, writing – review and editing. **Roghayeh Yahyazadeh:** data curation, formal analysis, investigation, methodology, resources, supervision, validation, visualization, writing – review and editing.

## Funding

The authors have nothing to report.

## Ethics Statement

According to the policies of the review board of our institute, single case reports are not subject to the requirement of ethical committee approval.

## Consent

Written informed consent was obtained from the patient for participation in this intervention and publication of associated clinical data with the understanding that this information is publicly available. To ensure confidentiality, all patient information was anonymized.

## Conflicts of Interest

The authors declare no conflicts of interest.

## Data Availability

The data that support the findings of this study are available from the corresponding author upon reasonable request.
